# Co-production as an approach for developing youth advocacy videos on countering unhealthy food marketing and e-cigarette advertising

**DOI:** 10.1186/s12889-025-24095-z

**Published:** 2025-08-30

**Authors:** Marissa J. Smith, Caroline Vaczy, Shona Hilton

**Affiliations:** 1 School of Health and Wellbeing, University of Glasgow, Glasgow, G12 8TB UK; 2 Department of Social Work and Social Policy, University of Strathclyde, Glasgow, G4 0LT UK

**Keywords:** Co-production, Participatory research, Young people, Youth advocacy, Health promotion

## Abstract

**Background:**

Community-based participatory research has been a cornerstone of health promotion and public health for over two decades, providing a foundation for co-production approaches that actively involve communities in the design and implementation of interventions. This paper examines the application of co-production as a research approach for developing youth advocacy videos on countering unhealthy food marketing and e-cigarette advertising.

**Methodology:**

The research to co-produce videos was conducted with 33 young people aged between 12 and 16 living in the Central Belt of Scotland. The research comprised four stages: workshops, photovoice, focus groups and video development.

**Results:**

The findings presented are derived from the co-production process involved in creating two youth advocacy videos on unhealthy food marketing and e-cigarette advertising. Through this approach, young co-researchers were provided with opportunities to both learn about and conduct research, fostering their active involvement at every stage of the project. Co-production with young co-researchers enhanced the relevance and impact of advocacy videos, empowering participants through authentic collaboration. While challenges like power dynamics, engagement, and ethical considerations arose, fostering trust, inclusivity, and skill-building ensured success. The project highlighted the importance of youth-led approaches in creating relatable content and developing leadership and practical skills for broader applications. The advocacy videos produced in this study emphasise the critical importance of engaging young co-researchers in the research process. They highlight the necessity of safeguarding young people from harmful advertising practices while empowering them to take an active role in shaping health interventions.

**Conclusion:**

This research underscores the value of co-production in health promotion, demonstrating its effectiveness in tailoring interventions to the unique perspectives and needs of the target audience. Notably, it also addresses a significant gap in the existing evidence base, which has historically focused on individual interventions rather than systemic or community-level approaches.

**Supplementary Information:**

The online version contains supplementary material available at 10.1186/s12889-025-24095-z.

## Background

Advocates of evidence-based medicine and other health professionals have increasingly aimed to conduct research that identifies and addresses the priorities of patients and the public, a stance long recognised by social scientists as important [1–3]. Involvement at both individual and community levels is now widely considered a crucial component of research, as studies influenced or led by patients or citizens are more likely to yield results that are both acceptable and applicable [4]. This participatory approach is increasingly known as “co-production,” a multifaceted concept with its origins in and applications across various disciplines [5, 6], though it is sometimes viewed as somewhat ambiguous. In health research, co-production typically entails collaboration between researchers, health professionals, and patients, carers, or citizens, who bring unique perspectives and knowledge to the research process [5, 7]. By integrating the lived experiences of ‘experts by experience’ with the expertise of professionals, researchers can more effectively design studies that better address the questions that matter most to those who use services, improve the acceptability of interventions, and gain deeper insight into the outcomes that are most valued [8].

The increased uptake of co-production in health research is regarded as a positive move towards the democratisation of public and personal decision-making. It represents a shift away from traditional top-down models of research and policymaking, where decisions are often made without sufficient input from the individuals and communities affected by them. Although its success depends on the inclusion of diverse perspectives [9, 10]. Co-production has the potential to redefine traditional power dynamics, creating opportunities for researchers to engage in new forms of knowledge production and exchange [11]. Paterson-Young [12] argues that advocacy can play a crucial role in co-production by challenging and influencing the policies and positions of decision-makers through organised and systematic actions aimed at ensuring equitable outcomes. Supporting participants, especially young people, in taking an active role in co-production fosters not only opportunities for meaningful engagement but also the development of leadership skills. By empowering young people to become leaders in the co-production process, research can tap into fresh perspectives and innovative ideas that reflect the diverse needs and experiences of the population [12].

As a participatory research approach, co-production builds on the long-standing tradition of community-based participatory research, which has been widely applied in public health for over two decades [13, 14]. While co-production is increasingly used in health research, it is conceptually rooted in and closely aligned with participatory traditions such as participatory action research and community-based participatory research, which similarly emphasise collaboration, shared power, and community engagement. Co-production has gained significant traction in various fields, especially in public health, where it is employed to involve communities in the design and implementation of interventions [9]. It requires researchers to work in partnership with participants, sharing power and decision-making responsibilities to produce outcomes that are directly informed by the experiences and insights of those participants. In the field of health promotion, co-production has been shown to improve the relevance and effectiveness of interventions by ensuring they are tailored to the specific needs and preferences of the target audience [15, 16]. However, co-production also presents challenges, such as managing power dynamics, ensuring meaningful participation, and addressing ethical concerns [10, 17]. Efforts at co-production can also fall short if findings that align with researchers’ existing conceptions are prioritised while other perspectives are dismissed or ignored [9].

The present study was driven by the growing influence of the commercial determinants of health (CDoH), particularly how marketing strategies employed by commercial entities shape the health behaviours and perceptions of young people. With the pervasive reach of commercial entities in promoting unhealthy products, we sought to explore how these influences affect young people’s decision-making and well-being. Recognising that these commercial forces are often unevenly distributed across socioeconomic groups [18], we also aimed to examine how such influences exacerbate health inequalities, disproportionately impacting young people from disadvantaged backgrounds. The research aimed to engage young people directly in analysing and responding to these commercial tactics, recognising that their perspectives should be central to developing effective health advocacy and policy interventions. By employing elements of a co-production approach, the study sought to highlight the broader impacts of CDoH on young people’s health, health and well-being, while empowering them to address these inequalities and advocate for healthier, more equitable communities.

## Methodology

This paper primarily serves as a reflection on the application of co-production as a participatory research approach in developing youth advocacy videos on unhealthy food marketing and e-cigarette advertising, thus, it is essential to discuss the methodological approach adopted during the study. The following sections outline the sampling and recruitment of the young co-researchers and the research design.

### Sampling and recruitment

The study purposively sampled 33 young co-researchers aged 11–16 from the Central Belt of Scotland, representing diverse socioeconomic backgrounds. Co-researchers were recruited through local youth organisations and schools, with youth workers facilitating communication and distribution of information. Co-researchers were required to complete consent forms and a brief questionnaire on their demographic information and their general experiences with food and e-cigarette marketing. Prior to participation, all young co-researchers provided informed consent, with parent/guardian consent also obtained for those under 16.

The composition of workshop and focus group sessions was determined by youth workers and school staff who acted as gatekeepers. We relied on their knowledge of the young people to form groups they felt would be most conducive to open and respectful discussions. As a result, some sessions included co-researchers from both the most and least deprived socioeconomic areas, however we did not feel this was a concern as the young people all knew each other from outside the workshops and focus groups. While we were aware of the potential sensitivities that can arise in mixed-SES groups, we did not observe any discomfort or reluctance to participate. To support a safe and inclusive environment, facilitators reiterated at the start—and throughout each session—that the space was confidential, that all contributions were valued, and that mutual respect was essential. These measures helped ensure that all co-researchers felt comfortable sharing their views and experiences.

### Research design

The co-production approach used in this study was designed to actively involve young co-researchers throughout the research process. This approach is rooted in the principles of participatory action research [19], where participants are considered co-researchers rather than subjects of the research. The co-production process was structured into four key stages: workshops, photovoice elicitation, focus groups, and video production. The four-step co-production process used in this study was developed specifically to support iterative engagement and youth-led knowledge generation. While not directly adapted from a single existing framework, the structure was informed by principles of participatory action research [19] and community-based participatory research [13], both of which emphasise collaboration, reflection, and shared ownership. The sequence was designed to build progressively: workshops introduced co-researchers to the project and generated initial ideas; photovoice enabled participants to document their lived experiences; focus groups facilitated deeper exploration and interpretation of those experiences; and video production translated insights into advocacy outputs. This structure allowed for continuous feedback loops and ensured that co-researchers’ voices shaped the project at every stage, aligning with the core values of co-production.

#### Stage 1: Workshops

The initial stage involved a series of three workshops held from March to April 2023, involving 33 co-researchers (10–12 per group), designed to introduce co-researchers to the concept of co-production and to generate initial ideas for the advocacy video. Prior to the workshops, the research team developed a structured facilitation guide informed by participatory research principles and youth engagement best practices. Facilitators received advice from other expert researchers who work with young people and training on co-production values, inclusive facilitation techniques, and safeguarding protocols to ensure a supportive and empowering environment for the co-researchers. Youth workers and school staff played a key role in recruitment and communication, helping to distribute information sheets and consent forms, and supporting the orientation process. As mentioned in the recruitment section, co-researchers were provided with detailed information outlining the project’s aims, their role as co-researchers, and what to expect during the project workshops. These preparatory steps were essential in building trust, ensuring informed participation, and laying the groundwork for meaningful engagement throughout the project.

To support relationship-building and help co-researchers feel comfortable in the research setting, each workshop began with informal introductions and icebreaker activities. These included conversational group exercises designed to encourage interaction and foster a sense of connection among co-researchers and between the co-researchers and the research team. These activities played a key role in establishing a welcoming and inclusive environment, which was essential for promoting open dialogue and active engagement throughout the project. We then moved on to discuss unhealthy food marketing, followed by e-cigarette advertising through a series of activities. The workshops were highly interactive and included brainstorming sessions and group discussions, to encourage co-researchers to share their thoughts and experiences related to food and e-cigarette marketing (Appendix A). The workshops also served as a space for co-researchers to begin thinking critically about the ways in which advertising influences their perceptions and behaviours. Researchers facilitated these sessions, with the co-researchers identifying key themes and messages that could be conveyed in the video. Each workshop lasted approximately two hours. The workshops served as a foundation for the subsequent stages, providing valuable insights into young co-researchers’ perceptions and concerns regarding unhealthy food marketing and e-cigarette advertising, as well as their visions for the advocacy videos. Each co-researcher received a £20 shopping voucher as compensation for their time. The workshops were not recorded in an effort to facilitate the comfort of co-researchers. Instead, researchers took field notes, and materials produced were kept for analysis. Researchers conducted initial coding of the material and notes produced from the workshops, identifying recurring themes related to perceptions of advertising, emotional responses, and perceived impacts. Particular attention was paid to differences in how these themes manifested across groups from the most and least deprived areas, as defined by SIMD rankings.

Thirty-three co-researchers aged 12–16 participated in this initial stage of the study (20 females (61%) and 13 males (39%)). Table 1 describes the workshop composition by area of deprivation. Deprivation rank was assigned using the Scottish Index of Multiple Deprivation (SIMD) [20]. The area-level deprivation was grouped using a binary deprivation variable (least deprived/most deprived) in which the three most deprived quintiles were grouped into the most deprived category for the Central Belt of Scotland area. This grouping was informed by the understanding that commercial influences are often distributed unevenly across different socioeconomic groups [18], contributing to greater health disparities that tend to disproportionately affect disadvantaged young people.

**Table 1:** Workshop location, composition and co-researcher details

Group	Area	Sex	Age
1	Most deprived (7) Least deprived (5)	Mixed: male (1)/female (11)	12–16
2	Most deprived (1) Least deprived (10)	Mixed: male (6)/female (5)	14–16
3	Most deprived (9) Least deprived (1)	Mixed: male (6)/female (4)	14–15

#### Stage 2: Photovoice

In the second stage, co-researchers were tasked with capturing photos or short videos of food and e-cigarette advertisements they encountered in their daily lives. This method aligns with the principles of photovoice, a participatory research approach that empowers participants to document and reflect on their lived experiences through photography [13, 21]. Originally developed by Wang and Burris [13] photo voice is grounded in community-based participatory research and included an advocacy component, making it particularly well-suits to youth led health promotion initiatives. Co-researchers were provided with guidance on how to capture relevant photos and videos during the workshop stage (Stage 1), including ethical and privacy considerations. This guidance was reiterated in a follow-up email sent to gatekeepers after the workshops to ensure consistency and support for co-researchers. The images collected by the co-researchers highlighted the pervasive presence of food and e-cigarette advertising in their environments and were instrumental in shaping the narrative of the advocacy videos. These images served as discussion prompts in the focus groups, helping to deepen the understanding of young co-researchers’ experiences and informing the development of the video. Of the 33 co-researchers involved in Stage 1, 32 submitted photos for Stage 2. Upon receipt of the photos, co-researchers were given a £20 voucher as compensation for their time.

#### Stage 3: Focus groups

The third stage involved conducting six focus groups, each consisting of five to six co-researchers (a total of 28 co-researchers). The focus groups were designed to delve deeper into the themes identified during the Stage 1 workshops and to discuss the photos and videos captured independently by the co-researchers in Stage 2, which we displayed during discussions via a slideshow. The discussions explored the impact of food and e-cigarette advertising on young co-researchers’ perceptions and behaviours, with a particular focus on how these advertisements were perceived by people in their age group (Appendix B). As with the workshops, focus groups included dedicated time for both unhealthy food marketing and e-cigarette advertising. Discussions typically began with food marketing before transitioning to e-cigarette advertising, enabling co-researchers to reflect on each topic individually and draw connections between them. Groups lasted between 48 and 75 min. Each co-researcher received a £20 shopping voucher as compensation.

The focus group transcripts were analysed thematically using Braun and Clarke’s six-phase approach to thematic analysis [22]. This method was selected due to its capacity to reveal both commonalities and contrasts in co-researcher responses, as well as its openness to unexpected findings [22, 23]. Members of the research team engaged in repeated readings of the transcripts to immerse themselves in the data. A coding framework was then developed collaboratively by two researchers, combining deductive themes aligned with the study’s objectives and inductive themes that emerged organically from the data (see Appendix C). Transcripts were uploaded into NVivo V.12 and independently coded by MJS and CV. The coding was then cross-checked, and discrepancies or divergent cases were discussed in light of both the transcripts and accompanying field notes to ensure a nuanced interpretation of the data. As with the workshops, researchers paid attention to differences in how themes manifested across groups from the most and least deprived areas. These thematic differences (from both the workshops and focus groups) were discussed in follow-up sessions with co-researchers, who were invited to reflect on and validate the emerging interpretations. This iterative feedback process helped ensure that the final advocacy videos authentically represented the diverse perspectives of the co-researchers involved, and that socioeconomic context was meaningfully embedded in the narrative structure and messaging.

Of the 33 initial co-researchers, 28 young co-researchers aged 12–16 (16 females (57%) and 12 males (43%)) participated in this stage of the study. The sample was slightly skewed towards 14–15-year-olds, with 14-year-olds being the largest subgroup (*n* = 12). Table 2 outlines the composition of the focus groups.

**Table 2:** Focus group location, composition and co-researcher details

Group	Area	Sex	Age
1 A	Most deprived (2) Least deprived (2)	Female (4)	12–15
1B	Most deprived (1) Least deprived (2)	Female (3)	13–16
2 A	Least deprived (5)	Mixed: male (3)/female (2)	14–16
2B	Most deprived (5) Least deprived (1)	Mixed: male (3)/female (3)	14–16
3 A	Most deprived (5)	Mixed: male (4)/female (1)	14–15
3B	Most deprived (4) Least deprived (1)	Mixed: male (2)/female (3)	14–15

#### Stage 4: Video production

The final stage involved the collaborative development of the advocacy videos with a professional video production company, Media Co-Op. A series of three sessions were organised by the researchers and were attended by a subgroup of four co-researchers, two members of the research team, and two facilitators from the video production company. The first session focused on reviewing draft scripts and visual concepts developed from earlier stages. The second session involved feedback on storyboarding and stylistic elements, including tone, colour palette, and pacing. The final session was a review of the near-final cut, where co-researchers provided final input on voiceover delivery and visual sequencing. Throughout this process, co-researchers’ input was prioritised and directly integrated into the creative decisions. The research team acted as facilitators and mediators, ensuring that co-researchers’ ideas were respected while also drawing on the production company’s expertise to guide technical and narrative coherence. The videos aimed to convey key messages identified during the earlier stages, using engaging visuals and voiceovers provided by two co-researchers. The collaborative nature of this stage ensured that the final product authentically represented the voices of the co-researchers, making it a powerful tool for advocacy.

## Discussion

For the results pertaining to the two videos, please see Smith et al. [24] and Smith et al.

Final versions of the two videos are available:

E-cigarette advertising: https://www.youtube.com/watch?v=xwZ-aoQIwjs&t=39s.

Unhealthy food marketing: https://www.youtube.com/watch?v=uXgV4T8X7DQ.

This section reflects our experience of co-production in this study, analysing its benefits, challenges and broader implications for research and advocacy. We will highlight both the facilitators and barriers encountered during the process, exploring the strategies that helped foster trust, engagement, and active participation among the young co-researchers, as well as the logistical or social challenges that arose. Additionally, we will examine the broader value of co-production, including how it empowered young co-researchers by providing opportunities for personal development, skill-building and leadership while ensuring that their voices shaped the research outcomes. We will also assess how the co-production approach enhanced the relevance and authenticity of the advocacy videos, making them more impactful and aligned with the real concerns of the target audience. Through this reflection, we aim to provide a comprehensive understanding of how co-production can contribute not only to the specific outcomes of a project but also to the broader research agenda, helping shape future research practices and policy development.

### Benefits of co-production

The use of co-production in this study offered several key benefits, enhancing the relevance, authenticity and impact of the advocacy videos created. By incorporating the lived experiences of young co-researchers, the videos were informed by the issues and perspectives most pertinent to the target audience. This approach ensured that the content resonated more effectively and meaningfully with the viewers. Research shows that youth-led and participatory approaches are highly effective in addressing issues that affect young people because they empower participants and reflect their genuine concerns [25]. Throughout the project, each stage of the co-production process built upon the previous one, allowing insights to evolve and deepen. For example, during the initial workshops, co-researchers discussed their perceptions of tobacco companies, with one group describing them as “like a god” — a powerful, omnipresent force shaping behaviour through advertising. This metaphor resonated strongly and was later developed into a central narrative device in the e-cigarette advocacy video. Similarly, the photovoice stage revealed the ubiquity of unhealthy food advertising in everyday environments, which prompted deeper reflection during focus groups on how these messages target young people. These discussions directly influenced the tone and messaging of the final videos, ensuring they reflected the lived experiences and priorities of the co-researchers. This iterative process not only strengthened the authenticity of the outputs but also reinforced the youth co-researchers’ sense of ownership and agency throughout the project.

Meaningful collaboration with young people cultivates a stronger investment in initiatives, increasing the likelihood of creating impactful and relatable content. In this study, the young co-researchers were able to create advocacy materials that directly aligned with the realities and aspirations of their peers, which amplifies the potential for behavioural change or advocacy success [26, 27]. The co-produced advocacy videos were successfully launched during webinars attended by a diverse audience, including policymakers, third-sector organisations, and educators. Additionally, the videos were presented at several national conferences, further amplifying their reach and impact. The research team also reconnected with the young co-researchers after the completion of the videos to share the final products with them and hear their thoughts on the co-production process.

Co-production also encourages long-term impact by integrating the perspectives of young co-researchers. Advocacy efforts that incorporate these perspectives tend to resonate more deeply with young audiences, fostering credibility and a sense of empowerment [27, 28]. This empowerment can help young people develop skills in leadership, collaboration, and problem-solving, which can be applied beyond the specific project context [27, 28]. One of the most impactful aspects of the advocacy videos in our study was the inclusion of the co-researchers as narrators. This decision was crucial in ensuring that young audiences would connect more deeply with the content. The authentic voices of their peers made the message more relatable, authentic, and impactful, which speaks to the power of peer-to-peer communication in advocacy [27, 29]. For us, this experience has been a powerful reminder of the importance of listening to, learning from, and collaborating with young people to create research outcomes that are truly representative of their voices.

### Challenges and considerations

#### Power dynamics

One of the primary challenges in co-production research is addressing and managing power dynamics between researchers and participants. However, managing these dynamics within existing hierarchical structures proved challenging. Several of the workshops and focus groups were conducted in a classroom with a teacher or youth worker present, which, while beneficial in some respects, may have influenced the co-researchers’ willingness to express their views openly, due to the power structures in place. Navigating the tension between the perceived authority of educators and the aim to create a collaborative environment was difficult at times. To counteract this, we aimed to foster an environment of trust and openness, where co-researchers were encouraged to share their views without fear of judgment. This required constant awareness of the power dynamics at play and the need to ensure the environment remained supportive and non-intimidating, allowing young people to feel comfortable voicing their opinions and contributing fully to the project.

Minimising hierarchical structures was a fundamental approach to balancing power dynamics within the co-production process. Instead of positioning the co-researchers as passive subjects, we deliberately framed them as co-researchers, emphasising their central role in shaping the project [30]. This shift required careful planning and consistent reinforcement of the idea that co-researchers held significant control over the project. Recognising that power imbalances can hinder the participation and contribution of young people, we took steps to ensure that the young co-researchers felt empowered to contribute their ideas and take ownership of the project. For example, the co-researchers were empowered to make decisions regarding the content and direction of the advocacy videos, including crafting storylines and selecting colour schemes. While the media team provided guidance and professional input, we ensured that the co-researchers retained final authority over the creative process. This approach was integral in fostering a sense of ownership and pride in their work. It was essential that the final products not only reflected their perspectives but also conveyed their voices authentically, ensuring the videos were genuine representations of their concerns and values. This empowerment, which was central to the co-production process, not only led to more meaningful and relevant outcomes but also reinforced the young co-researchers confidence in their ability to influence and create impactful change.

#### Participant engagement

Maintaining participant engagement throughout the multi-stage process posed another challenge. The extended duration of the project required ongoing efforts to sustain motivation and involvement. Scheduling conflicts and changes within the youth cohort contributed to the loss of some co-researchers over time. Though losing participants is never desirable and is always an unfortunate reality in long-term research, this experience underscored the critical importance of designing research projects that align with the real-life commitments of young people, such as school, extracurricular activities and personal obligations. Working with youth organisations and schools also underscored the value of engaging with group representatives to streamline communication and logistics. While this approach had its benefits, it also posed challenges in maintaining direct connections with all participants. Although working through group representatives can enhance the logistical efficiency of a project, it sometimes inadvertently distances the researchers from individual participants, which can impact the sense of personal connection and ownership. Beyond working with gatekeepers, several strategies helped sustain engagement in our project. Providing flexibility in how and when young people could participate—such as asynchronous activities (e.g., stage 2: Photovoice) and online options—proved effective. Creating a strong sense of ownership by valuing their ideas, ensuring their contributions had visible impact, and incorporating creative, interactive methods also helped maintain motivation. These experiences highlight the need for flexibility, adaptability, and ongoing communication in participatory research, especially when working with young people. They also underscore the importance of regular check-ins and open feedback channels to ensure that participants remain engaged and feel supported throughout the process.

#### Ethical considerations

Research involving young people necessitates careful attention to ethical considerations, particularly concerning informed consent and participant well-being. In this study, the research team adhered to rigorous ethical guidelines to ensure that co-researchers fully understood the project’s aims, their roles, and their rights. From the outset, we made it a point to engage in clear and transparent communication, ensuring that the co-researchers were not only informed but also empowered to ask questions and seek clarification about their involvement at any point during the study.

To safeguard the well-being of co-researchers, we prioritised creating a supportive and respectful environment throughout the project. In addition to obtaining informed consent from both co-researchers and their parents or guardians, we implemented several measures to ensure co-researcher safety and comfort. Regular informal check-ins were conducted with co-researchers during each stage of the study to ensure they felt supported and able to raise any concerns. As outlined in the study’s information sheets and consent forms, both co-researchers and their parents or guardians were encouraged to contact the research team at any time with questions or concerns. Youth workers or teachers who were known and trusted by the co-researchers were present at all stages and were available to offer support if needed. Clear communication regarding expectations, consent, and the nature of the study was prioritised throughout, ensuring that co-researchers always understood their rights and how their input would be used. Additionally, we maintained open channels for feedback, where co-researchers could share any discomfort or questions that arose, and offered access to resources or additional assistance if needed. By keeping these ethical practices at the forefront, we not only safeguarded the co-researchers’ well-being but also worked to build a foundation of trust. The co-researchers were encouraged to express their thoughts and concerns openly, knowing their feedback would be taken seriously and respected. This approach reinforced the co-researchers’ confidence in the research process, helping them feel more engaged and empowered throughout the project.

#### Budget considerations

Delivering a multi-stage co-production project of this nature required careful budgeting to ensure meaningful engagement and high-quality outputs. Key cost areas included co-researcher compensation, travel expenses, researcher time for facilitation and analysis, and professional services from the creative agency. The collaboration with Media Co-Op, which included various creative sessions and full video production, represented a significant portion of the budget. These considerations are essential for funders and practitioners aiming to implement co-production with young people in a similar way.

#### Balancing expertise and participation

While the co-production process prioritised youth voice and leadership, we recognise that effective public health communication also requires strategic, behavioural, and technical expertise. The involvement of Media Co-Op, a professional creative agency, was instrumental in translating co-researcher-generated ideas into compelling and technically sound advocacy videos. Their guidance ensured that the outputs were not only authentic but also visually engaging and aligned with best practices in health messaging. Although the co-researchers had final input on creative decisions, these were shaped and supported by expert facilitation to ensure feasibility and impact. We also acknowledge the limitations of relying on a small group of youth representatives to capture the full diversity of the target audience. Future projects may benefit from broader audience testing or iterative feedback loops to further refine messaging. Ultimately, we view co-production and expert input as complementary rather than substitutional—each essential to producing content that is both meaningful and effective.

#### Balancing process and product

While this study foregrounded the empowerment and skill development of young co-researchers, we recognise that the ultimate goal of public health advocacy is to deliver messages that are both compelling and effective in influencing behaviour. There is an inherent tension between prioritising participatory processes and ensuring the strategic impact of the final outputs. In our project, we sought to navigate this by integrating professional expertise—through collaboration with a creative agency—while maintaining youth leadership in shaping the narrative and tone of the videos. This partnership allowed us to uphold the authenticity of youth voices while ensuring technical quality and alignment with public health communication principles. Future co-production initiatives may benefit from explicitly planning for this balance from the outset, incorporating iterative testing and audience feedback to refine outputs without compromising the participatory ethos. Acknowledging this dual imperative—process and product—can help ensure that co-produced materials are not only empowering for participants but also effective tools for broader public health impact.

### Reflections on co-production with young people

#### Building trust and relationships

Our co-production approach was grounded in the importance of relationship-building, which was central to ensuring that the young co-researchers felt both comfortable and empowered throughout the process. From the outset, we recognised that establishing a foundation of trust would be pivotal in creating an environment where co-researchers felt safe to share their ideas and perspectives. This trust-building process started with initial introductory workshops, where the young co-researchers were not only familiarised with the project’s aims but also introduced to the research team and the collaborative nature of the work. During these workshops, the setting and layout allowed researchers to engage with smaller groups of co-researchers, facilitating more detailed discussions about the project. This structure created opportunities to share personal experiences and reflections on discussed topics (e.g., exposure to unhealthy food marketing). These early sessions set the tone for the entire project, allowing co-researchers to get to know the researchers in a relaxed, informal setting. These efforts at relationship-building were not just about creating a comfortable working environment but also about laying the groundwork for authentic collaboration. As a result, the co-researchers were more likely to invest personally in the project, taking ownership of their contributions and feeling a deeper sense of connection to the outcomes. Creating this open, collaborative atmosphere helped to ensure that the voices and perspectives of the co-researchers were genuinely integrated into the project, which ultimately enriched the overall process and its outcomes [31, 32].

#### Recognising diverse perspectives

Young people are not a homogenous group, and their experiences, views, and priorities can vary widely. Acknowledging and addressing this diversity is crucial in co-production. Our methods included diverse recruitment strategies to ensure representation from different backgrounds and interests. Moreover, our approach was tailored to respect individual differences and encourage open dialogue. Recognising that not all young people would feel comfortable expressing themselves in large group settings, we implemented strategies such as focus groups. These smaller, focused discussions allowed co-researchers to explore issues in more depth, share their thoughts freely, and engage in a more intimate and supportive environment. This format also created opportunities for voices that might typically be overshadowed in larger group settings to be heard and valued. In turn, this inclusive approach enriched the project, ensuring that the advocacy efforts were relevant and meaningful to a broader and more diverse audience. The voices of young people from different backgrounds and experiences helped to shape materials that were not only impactful but also deeply connected to the realities faced by their peers. This approach helped us to create advocacy resources that truly resonated with the diverse communities of young people they aimed to serve, making them more effective in driving change and raising awareness about critical issues.

#### Capacity building and skill development

Co-production is not only about producing outcomes but also about empowering participants and enabling them to actively shape the process and the results. Our methods incorporated skill-building activities, such as workshops on digital storytelling, visual design and media production. In particular, the project supported the development of both functional (practical skills needed to use media) and critical media literacy (analysing, evaluating, and critiquing media content) [33]. Co-researchers engaged with media not only as consumers—by analysing advertising techniques and their effects—but also as creators, by producing advocacy content. This dual engagement helped them build practical skills in scripting, storyboarding, and voiceover work, while also fostering critical thinking about how media messages are constructed, targeted, and interpreted. These experiences encouraged co-researchers to question the intent and impact of commercial advertising, particularly in relation to their own health and well-being. These opportunities enabled co-researchers to contribute confidently and effectively while also equipping them with skills they could apply in other areas of their lives. These workshops not only helped co-researchers develop technical skills but also nurtured their creativity, problem-solving abilities, and confidence in their own capabilities. A key moment in the process was when the co-researchers who narrated the videos visited a media production company. This experience gave them valuable insights into the media production process and helped them understand how their voices would be incorporated into the final product. We witnessed a growing sense of ownership and pride among co-researchers as they began to see their input reflected in the final product. The co-researchers were not just passive contributors; they were active co-researchers in crafting the story, shaping the content, and making key decisions about how the videos would be presented. This process empowered them to take ownership of the project and feel invested in its success. In addition to the practical skills they gained, co-researchers also reflected positively on the experience of developing skills that extended beyond the immediate project context. Co-production, when done with a focus on empowerment and skill development, is a powerful tool for equipping young people with the tools and confidence they need to drive change in their own lives and communities [34].

#### Reflective practice for researchers

A critical component of our co-production approach was the researchers’ commitment to continuous self-reflection throughout the process. We understood that co-production is not a static process but one that requires flexibility, adaptability, and ongoing evaluation to ensure its success. We regularly reflected on our methods, assumptions, and interactions with co-researchers to ensure that the process remained truly participatory and responsive to the needs of the young co-researchers. This practice of reflection allowed us to adapt our strategies, make necessary adjustments, and improve our approach as the project evolved.

Regular debriefs and feedback sessions with the co-researchers were central to this reflective practice. These sessions provided us with invaluable insights into the experiences of the young co-researchers, allowing them to share their perspectives on the process, offer suggestions for improvement, and express any challenges they had encountered. Importantly, these sessions helped us identify areas where we could strengthen our support and engagement, as well as aspects of the project that were particularly successful and should be carried forward in future initiatives. One key lesson that surprised us was the extent to which young people valued creative control and the ability to make meaningful decisions about the videos. This challenged our initial assumptions about the balance between guidance and independence in co-production, reinforcing the importance of allowing young people to take the lead in shaping both the process and outcomes.

Engaging in ongoing dialogue with the co-researchers not only allowed us to refine our approach but also reinforced the sense of ownership and agency among the co-researchers. By actively involving them in the evaluation of the process, we ensured that their voices were consistently integrated into decision-making. This feedback loop helped maintain a high level of trust and collaboration, as it demonstrated our commitment to creating an environment This reflective practice was crucial in ensuring that co-production remained inclusive, empowering, and responsive to the needs of all involved. It also provided us with the tools to evaluate the effectiveness of our methods and identify ways to improve in future co-production projects. Through this process of mutual reflection and adaptation, we were able to continually refine our approach, ensuring that co-production remained at the heart of the research and advocacy work [32].

#### Campaign reach and evaluation

Following the completion of the co-production process, the two advocacy videos were disseminated through webinars, social media platforms, and national and international conferences. The launch webinars were attended by approximately 85 participants, including educators, public health professionals, and third-sector organisations. While this study did not include a formal post-distribution evaluation of the advocacy videos, we recognise the importance of such assessment and view it as a valuable direction for future research. Following dissemination through webinars, social media, and national and international conferences, informal feedback indicated that the videos were well received by a range of relevant stakeholders. Although these indicators suggest promising engagement, a structured post-distribution evaluation involving young people, educators, and community stakeholders would provide deeper insights into the videos’ impact, relevance, and effectiveness. We recommend incorporating such evaluations in future co-produced advocacy projects to better understand audience engagement and inform the development of similar initiatives. Additional insights into the campaign’s reach and effectiveness are available in two related publications: a publicly accessible report on youth perceptions of unhealthy food advertising [24] and a peer-reviewed article on the e-cigarette video [35].

## Conclusion

Co-production offers a powerful and inclusive approach for engaging young people in research and advocacy, ensuring that their voices are not only heard but are also meaningfully integrated into the outcomes of the research process. This approach transforms young people from passive subjects into active contributors, empowering them to influence and shape the direction of the work, ultimately leading to more relevant and impactful findings. The advocacy videos developed in this study exemplify the potential of this approach, demonstrating how young people can play an active role in addressing critical issues such as unhealthy food marketing and e-cigarette advertising. Beyond amplifying their voices, this approach equips young people with valuable research, critical thinking, and advocacy skills, contributing to their personal development and long-term empowerment as health champions. Future research should continue to explore and refine co-production methodologies, focusing on strategies that enhance inclusivity, overcome barriers to participation, and sustain engagement over time. By prioritising the active involvement of young people, co-production not only enriches the quality and relevance of research outcomes but also fosters a sense of ownership and agency among co-researchers, ultimately driving more impactful and sustainable change.

## Supplementary Information

Supplementary Material 1.

Supplementary Material 2.

Supplementary Material 3.

## Data Availability

No datasets were generated or analysed during the current study.

## References

[1] Chalmers I, Bracken MB, Djulbegovic B, Garattini S, Grant J, Gülmezoglu AM, et al. How to increase value and reduce waste when research priorities are set. Lancet. 2014;383(9912):156–65. 24411644 10.1016/S0140-6736(13)62229-1

[2] Popay J, Williams G. Public health research and lay knowledge. Soc Sci Med. 1996;42(5):759–68. 8685744 10.1016/0277-9536(95)00341-x

[3] Hughes R. Patient safety and quality: an evidence-based handbook for nurses. Rockville (MD): Agency for Healthcare Research and Quality; 2008. 21328752

[4] Arumugam A, Phillips LR, Moore A, Kumaran SD, Sampath KK, Migliorini F, et al. Patient and public involvement in research: a review of practical resources for young investigators. BMC Rheumatol. 2023;7(1):2. 36895053 10.1186/s41927-023-00327-w PMC9996937

[5] Filipe A, Renedo A, Marston C. The co-production of what? Knowledge, values, and social relations in health care. PLoS Biol. 2017;15(5):e2001403. 28467412 10.1371/journal.pbio.2001403 PMC5414996

[6] Williams SJ, Caley L. Defining Co-production. Improving healthcare services: coproduction, codesign and operations. Cham: Springer International Publishing; 2020. pp. 39–54.

[7] Needham C, Carr S. Co production: An Emerging Evidence Base for Adult Social Care Transformation. Social care institute for excellence; 2009.

[8] Poland F, Charlesworth G, Leung P, Birt L. Embedding patient and public involvement: managing tacit and explicit expectations. Health Expect. 2019;22(6):1231–9. 31538704 10.1111/hex.12952 PMC6882252

[9] Smith H, Budworth L, Grindey C, Hague I, Hamer N, Kislov R, et al. Co-production practice and future research priorities in United Kingdom-funded applied health research: a scoping review. Health Res Policy Syst. 2022;20(1): 36. 35366898 10.1186/s12961-022-00838-x PMC8976994

[10] Egid BR, Roura M, Aktar B, Amegee Quach J, Chumo I, Dias S, et al. ‘You want to deal with power while riding on power’: global perspectives on power in participatory health research and co-production approaches. BMJ Glob Health. 2021. 10.1136/bmjgh-2021-006978. 34764147 10.1136/bmjgh-2021-006978 PMC8587355

[11] Durose C, Perry B, Richardson L. Is co-production a ‘good’ concept? Three responses. Futures. 2022;142: 102999.

[12] Paterson-Young C, Adhikari J, Lee L, Maher M, Wright LHV. Creating ownership: strengths and tensions in co-production with children, young people, and adults across contexts. Childhood. 2024;0(0): 09075682241266814.

[13] Wang C, Burris MA. Photovoice. Concept, methodology, and use for participatory needs assessment. Health Educ Behav. 1997;24(3):369–87. 9158980 10.1177/109019819702400309

[14] Baker TA, Wang CC. Photovoice: use of a participatory action research method to explore the chronic pain experience in older adults. Qual Health Res. 2006;16(10):1405–13. 17079801 10.1177/1049732306294118

[15] Green LW, O’Neill M, Westphal M, Morisky D. The challenges of participatory action research for health promotion. Promot Educ. 1996;3(4):3–5. 9081644 10.1177/102538239600300401

[16] De Rosis S, Pennucci F, Noto G, Nuti S. Healthy living and co-production: evaluation of processes and outcomes of a health promotion initiative co-produced with adolescents. Int J Environ Res Public Health. 2020. 10.3390/ijerph17218007. 33143198 10.3390/ijerph17218007 PMC7662519

[17] Turnhout E, Metze T, Wyborn C, Klenk N, Louder E. The politics of co-production: participation, power, and transformation. Curr Opin Environ Sustain. 2020;42:15–21.

[18] Allen LN. Commercial determinants of global health. Handb Global Health. 2021;1275:310.

[19] Baum F, MacDougall C, Smith D. Participatory action research. J Epidemiol Commun Health. 2006;60(10):854–7. 10.1136/jech.2004.028662 PMC2566051 16973531

[20] Scottish Government. The Scottish Index of Multiple Deprivation 2020 2020. Available from: https://www.gov.scot/collections/scottish-index-of-multiple-deprivation-2020/.

[21] Shaw PA. Photo-elicitation and photo-voice: using visual methodological tools to engage with younger children’s voices about inclusion in education. Int J Res Method Educ. 2021;44(4):337–51.

[22] Braun V, Clarke V. Conceptual and design thinking for thematic analysis. US: Educational Publishing Foundation; 2022. pp. 3–26.

[23] King N. Using Templates in the Thematic Analysis of Text. 2004. pp. 257– 70.

[24] Smith MJ, Vaczy C, Kellerman A, Tulloch L, Hilton S. Young people’s perceptions of and exposure to unhealthy food advertising in Scotland. Scotland, UK: Scottish Obesity Alliance; 2024.

[25] Smith KE, Acevedo-Duran R, Lovell JL, Castillo AV, Cardenas Pacheco V. Youth are the experts! Youth participatory action research to address the adolescent mental health crisis. Healthcare. 2024;12(5): 592. 38470702 10.3390/healthcare12050592 PMC10930985

[26] Harris A, Wyn J, Younes S. Beyond apathetic or activist youth:‘ordinary’ young people and contemporary forms of participation. YOUNG. 2010;18(1):9–32.

[27] Spencer G, Fairbrother H, Thompson J. Privileges of power: authenticity, representation and the problem of children’s voices in qualitative health research. Int J Qual Methods. 2020;19. 10.1177/1609406920958597

[28] Checkoway B. What is youth participation? Child Youth Serv Rev. 2011;33(2):340–5.

[29] London JK, Zimmerman K, Erbstein N. Youth-Led research and evaluation: tools for youth, organizational, and community development. New Dir Evaluation. 2003;2003(98):33–45.

[30] Lohmeyer BA. Keen as fuck’: youth participation in qualitative research as ‘parallel projects. Qual Res. 2020;20(1):39–55.

[31] Hart R. Children’s Participation: From Tokenism To Citizenship. Innocenti Essays. 1992;4. URL: https://www.researchgate.net/publication/338535687_Privileges_of_Power_Authenticity_Representation_and_the_Problem_of_Children's_Voices_in_Qualitative_Health_Research

[32] Mann A, Liley J, Kellett M, Engaging, Young People in Research. Children and. 2014. In: Understanding Research with Children and Young People. SAGE Publications. 285-304.

[33] Chen D-T, Wu J, Wang Y-m. Unpacking new media literacy. J Systemics Cybernetics Inf. 2011;9(2):84–8.

[34] Aston HJ, Lambert N. Young people’s views about their involvement in decision-making. Educ Psychol Pract. 2010;26(1):41–51.

[35] Smith MJ, Vaczy C, Hilton S. Co-production of a youth advocacy video on the harms of e-cigarette advertising in Scotland. Health Promot Int. 2025;40(2): daae097. 40037914 10.1093/heapro/daae097 PMC11879641

