# Supplementary material for: Co-production as an approach for developing youth advocacy videos on countering unhealthy food marketing and e-cigarette advertising
Source: BMC Public Health. 2025 Aug 30;25:2979. doi: 10.1186/s12889-025-24095-z (PMC12398034; doi:10.1186/s12889-025-24095-z)
Supplement: Supplementary file 2 — Supplementary Material 2. [file 12889_2025_24095_MOESM2_ESM.docx]

**Focus group guide for food and e-cigarette marketing**

Introduction

My name is [insert name]. Thank you very much for agreeing to talk to me.

- Run through checklist
- Reiterate that I am interested to hear their thoughts on the photos that they took for the videos and their and experiences of food and e-cigarette marketing.

**Food photos**

To produce the videos, we asked you to take photos of food adverts you see in your daily lives. First of all, I want to talk about the photos that you took.

**Show slides of participants photos.**

**For each participants photos ask the following questions.**

1. Out of the photos [participant] took, which one do you think is the most striking?
   1. PROMPT: Why?
   2. What is it about that photo that makes you say that?
   3. How does that photo make you feel?
   4. Ask about the colour. Is the photo showing any specific colours? Does this influence attraction?
2. Out of the photos you took, what one is most effective?
   1. PROMPT: Why?

**Advertising**

1. Do you think these images are advertising a product?
   1. PROMPT: How do you know this?
2. What do you think they are advertising?
   1. PROMPT: Product?
   2. PROMPT: Brand?
3. Why do you think the advert has been designed this way?
   1. PROMPT: What makes you say that?
4. Do you think these images are successful in selling their product?
5. Who do you think these adverts are targeted at?
   1. PROMPT: Why do you say that?
6. Do you see adverts a lot when you are out and about?
   1. If so, where do you see them?
   2. PROMPT: What about on social media?
   3. How does that make you feel?
   4. Does that influence your choices?

**Promotions**

Now we are going to move on to talk about promotions.

1. What do you think the purpose is of the promotions?
2. Do you think these promotions are successful in selling their product?
3. Do promotions influence your choices?
4. Where in a shop/supermarket have you seen promotions?
   1. PROMPT: At the front of the shop?
   2. PROMPT: at the checkout?
   3. PROMPT: Online?
   4. Do you think the location of certain items within the shop makes people buy them more or less?
5. Are there specific times of the year that you see promotions?
   1. PROMPT: Seasonal (e.g., Easter)
   2. Where are these usually located?
   3. Does this influence your choices?

**Branding**

Now we are going to move on to talk about branding.

1. What do you think the difference is between advertising and branding?
2. Could you provide some examples of brands?
   1. PROMPT: Can be non-food related in struggling
3. How do you recognise a brand?
   1. PROMPT: by its colour
   2. PROMOT: Logo?
   3. PROMPT: Design
4. Are there any food brands that you regularly/always choose (e.g., Coke vs. Pepsi)?
   1. PROMPT: What about these brands makes you choose them?

5. What do you think companies invest time and money in branding?

**Celebrity/influencers**

Having discussed advertising, promotions and branding of foods we are now going to discuss celebrities and influencers.

1. Would you be more inclined to buy a product that is being used or advertised/promoted by a celebrity/influencer you like?

- 1. PROMPT: Why or why not?

1. Why do you think celebrities/influencers would want to advertise food products?
2. Why do you think companies would pay celebrities/influencers to advertise their products?

**Final food-related remarks**

1. How do you think food products should be advertised/promoted?
   1. Do you want to see different types of ads (e.g., more healthy foods?
   2. Do you want to see less advertising/promotions in general?
2. Do you think there should be restrictions on the advertising/promotions of certain types of food/drinks?
   1. PROMPT: Why/why not?
   2. PROMPT: How would this affect different groups of people in different ways?
   3. PROMPT: How would this affect businesses?
   4. PROMPT: Are there any locations (in person or online) where marketing should/should not be restricted?
   5. PROMPT: If yes, how do you think these restrictions could be enforced?

**E-cigarette photos**

To produce the videos, we asked you to take photos of e-cigarette marketing that you see in your daily lives. Before we talk about your photos:

1. Why do you think people use e-cigarettes?

1. PROMPT: What about adults vs youths?

Now I want to talk about the photos that you took.

**Show slides of participants photos.**

**For each participants photos ask the following questions.**

1. Out of the photos [participant] took, which one do you think is the most striking?
2. PROMPT: Why?
3. What is it about that photo that makes you say that?
4. How does that photo make you feel?
5. Ask about the colour. Is the photo showing any specific colours? Does this influence attraction?
6. Out of the photos you took, what one is most effective?
   1. PROMPT: Why?

**Advertising**

1. Do you think these images are advertising a product?
   1. PROMPT: How do you know this?
2. What do you think they are advertising?
   1. PROMPT: Product?
   2. PROMPT: Brand?

3. What do you think about the use of colour in the adverts?

1. Do you think this has been done on purpose?
2. Do you think this influences peoples’ choices?
   - 1. PROMPT: Why?
3. Do you think these images are successful in selling their product?
4. Who do you think these adverts are targeted at?
   1. PROMPT: Why do you say that?
5. Do you see/notice adverts for e-cigarettes a lot when you are out and about?
   1. If so, where do you see them?
   2. PROMPT: What about on social media?
   3. How does that make you feel?
   4. Does that influence your choices?

**Promotions**

Now we are going to move on to talk about promotions of e-cigarettes.

1. What do you think the purpose is of the promotions?
2. Do you think these promotions are successful in selling their product?
3. Do promotions influence your/peoples choices?
4. Where have you seen promotions?
   1. PROMPT: At the front of the shop?
   2. PROMPT: at the checkout?
   3. PROMPT: Online?
   4. Do you think the location of e-cigarette promotions makes people buy them more or less?

**Branding**

1. Can anyone name any brands of e-cigarettes?

**Flavours**

1. When I saw ‘flavours of e-cigarettes’ what springs to mind?

2. Can anyone name any flavours of e-cigarettes?

2. Do you see some flavours as more appealing compared to others?

- 1. What ones and why?

3. Do you think there are any flavours that are more appealing to youths compared to adults?

1. What ones and why?

4. Why do you think flavourings are attractive to youths?

**Final e-cigarette remarks**

1. Do you think e-cigarette products should be allowed to be advertised/promoted?
2. How do you think e-cigarette products should be advertised?
3. Do you think there should or should not be warnings on e-cigarette adverts products?
   1. Why?

**Celebrity/influencers**

Having discussed advertising, promotions, branding and flavours of e-cigarettes we are now going to discuss celebrities and influencers.

1. Would you be more inclined to buy an e-cigarette that is being used or advertised/promoted by a celebrity/influencer?

- 1. PROMPT: Why or why not?

1. Why do you think celebrities/influencers would want to advertise e-cigarettes?
2. Why do you think companies would pay celebrities/influencers to advertise their products?

**Concluding remarks**

1. Do you have any remarks or want to mention anything we have discussed?

**Thank you very much for taking part in this focus group. I appreciate you taking the time to participate and for providing your thoughts.**
